# Regulatory networks of non-coding RNAs in brown/beige adipogenesis

**DOI:** 10.1042/BSR20150155

**Published:** 2015-10-30

**Authors:** Shaohai Xu, Peng Chen, Lei Sun

**Affiliations:** * Cardiovascular and Metabolic Disorders Program, Duke-NUS Graduate Medical School, 8 College Road, Singapore 169857, Singapore; † School of Nuclear Science and Technology, University of Science and Technology of China, 443 Huangshan Road, Hefei, Anhui 230027, P.R. China; ‡ Division of Bioengineering, Nanyang Technological University, 70 Nanyang Drive, Singapore 637457; § Institute of Molecular and Cell Biology, 61 Biopolis Drive, Proteos, Singapore 138673, Singapore

**Keywords:** brown/beige adipocyte, lncRNA, miRNA, obesity

## Abstract

BAT (brown adipose tissue) is specialized to burn fatty acids for heat generation and energy expenditure to defend against cold and obesity. Accumulating studies have demonstrated that manipulation of BAT activity through various strategies can regulate metabolic homoeostasis and lead to a healthy phenotype. Two classes of ncRNA (non-coding RNA), miRNA and lncRNA (long non-coding RNA), play crucial roles in gene regulation during tissue development and remodelling. In the present review, we summarize recent findings on regulatory role of distinct ncRNAs in brown/beige adipocytes, and discuss how these ncRNA regulatory networks contribute to brown/beige fat development, differentiation and function. We suggest that targeting ncRNAs could be an attractive approach to enhance BAT activity for protecting the body against obesity and its pathological consequences.

## BAT ADIPOGENESIS AND ITS MODULATORS

BAT (brown adipose tissue), a specialized mammalian organ, is distinguished from WAT (white adipose tissue) by its location, morphology and physiological function [1]. BAT promotes energy expenditure via uncoupled respiration mediated by mitochondrial UCP1 (uncoupling protein 1) [2]. Another type of adipocyte expressing UCP1 is referred as beige or brite (brown in white) adipocyte and regarded as a non-classical/inducible brown adipocyte [3,4]. Ablation of BAT can induce obesity and metabolic diseases [5]; in contrast, enhancing BAT activity and function through different strategies leads to increased energy expenditure against obesity [6]. Therefore manipulation of BAT mass and function is an attractive therapeutic approach for obesity-associated metabolic syndrome and its complications.

There has been an upsurge of interest in understanding the mechanism underlying brown/beige adipogenesis. The master regulator of brown fat development is PRDM16 (PR domain-containing 16) which specifies the brown fat lineage from white fat and muscle [7]. It drives brown adipocyte development by binding and activating the transcriptional cofactors PGC-1α [PPAR (peroxisome-proliferator-activated receptor) γ co-activator 1α], PGC-1β, PPARα and PPARγ to induce brown-fat-selective genes, and by interacting with transcriptional co-repressors such as CtBP1 (C-terminal binding protein 1) and CtBP2 to repress white-fat-cell-selective genes [8,9]. In addition, a variety of extrinsic or intrinsic protein factors have been identified as regulators for BAT adipogenesis and physiological function (Figure 1). These studies have been reviewed in other excellent articles [6,10–13] and so we will not discuss these extensively in the present review.

**Figure 1:**
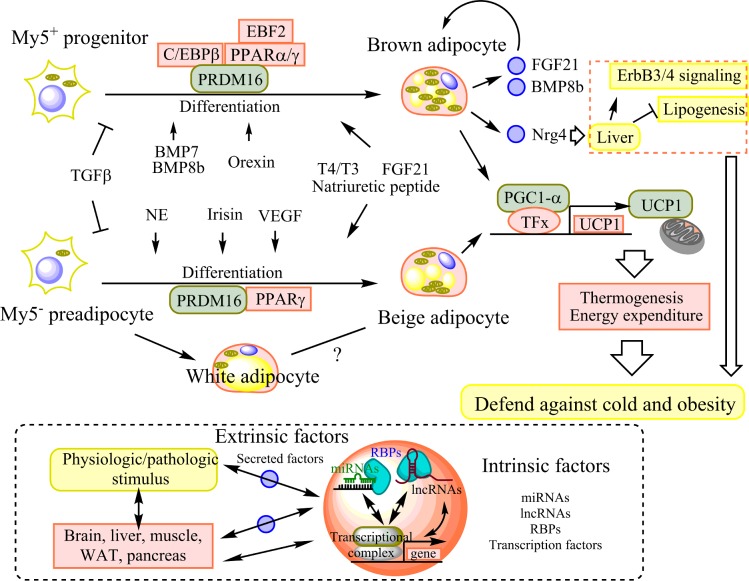
Key protein/ncRNA regulatory networks regulate brown/beige adipogenesis Brown and beige adipocytes originate from distinct progenitors, and are differently influenced either by extrinsic factors indirectly regulating key transcription factors (such as PRDM16 and PPARγ), or through mobilizing intrinsic factors (such as ncRNA). BAT executes its metabolic function mainly by fuel oxidation and UCP1-mediated thermogenesis to defend against cold and obesity. For detailed descriptions of these proteins and ncRNAs, see the text. BMP, bone morphogenetic protein; NE, noradrenaline (norepinephrine); RBP, RNA-binding protein; T3, 3,3′,5-tri-iodothyroninel; T4, thyroxine; TFx, thyroid receptor; VEGF, vascular endothelial growth factor.

In the present review, we focus on the function of ncRNA (non-coding RNA) in brown/beige fat development and consider two distinct subsets of ncRNAs: the 20–24-nt miRNA, and the lncRNA (long non-coding RNA), which is arbitrarily classified as RNA longer than 200 nt [14]. miRNA is evolutionarily conserved and recognizes its target mRNA by imperfect pairing between its seed sequence and mRNA. Paired miRNA–mRNA together with Argonaute protein form a RNA–protein complex, miRISC (miRNA-induced silencing complex), in which miRNA induces target mRNA translational repression and/or AGO2 (Argonaute 2)-mediated mRNA cleavage [15,16]. In contrast, lncRNA has poor sequence conservation, and through its primary, secondary and tertiary structures, interacts with RBPs (RNA-binding proteins) to regulate gene expression at transcriptional or post-transcriptional levels [14,17]. Transcriptome studies have revealed distinctive expression patterns of ncRNA in different adipose depots, a dynamic change in ncRNA expression during BAT and WAT adipogenesis and functional importance of these ncRNAs in these biological processes [14,18–20]. In the present review, we aim to cover the recent advances in understanding the role of ncRNAs in brown/beige adipocyte biology (Table 1 and Figure 1) and point out that manipulating ncRNAs could be a novel therapeutic approach for combating metabolic diseases. Because of limitations of space, it is impossible to cover all articles published in this rapid growing field, so we only focus on a few seminal pioneering studies and some recent studies with physiological evidence. We apologize for many excellent studies that are not discussed in the present review.

**Table 1:** miRNAs that regulate brown/beige fat cell adipogenesis and physiological function

miRNA	Expression profile	Function	Phenotype of miRNA transgenic or knockout mouse
*miR-193b-365*	BAT, WAT, other	Promotes brown adipogenesis	No transgenic mice
*miR-133*	Muscle, BAT, SAT	Inhibits brown/beige adipogenesis	Knockout mice: SAT browning, increased insulin sensitivity and glucose tolerance
*miR-196a*	BAT, SAT	Promotes beige adipogenesis	Transgenic mice: enhanced energy expenditure, resistance to obesity and the metabolic syndrome
*miR-155*	BAT, WAT	Inhibits brown/beige adipogenesis	Transgenic mice: reduced BAT mass; knockout mice: increased ability to adapt to cold exposure
*miR-27*	BAT, WAT	Inhibits brown/beige adipogenesis	No transgenic mice
*miR-378*	Muscle, BAT, WAT	Promotes brown adipogenesis	Transgenic mice: increased BAT mass, prevent both genetic and HFD-induced obesity
*miR-34a*	Liver, BAT, WAT	Inhibits brown/beige adipogenesis	No transgenic mice

## FUNCTION OF miRNAs IN BROWN/BEIGE FAT DEVELOPMENT AND PHYSIOLOGY

### DGCR8 or Dicer adipose-knockout impairs the function of BAT

To determine the role of miRNA, as a whole, in adipose tissue, several mouse strains of adipose-specific knockout of DGCR8 (DiGeorge syndrome critical region 8) or Dicer, two key regulators of miRNA biogenesis pathways, have been generated [18,21]; in addition, a tamoxifen-inducible Dicer-knockout strain has also been generated [21]. Both DGCR8- and Dicer-knockout mice display enlarged but pale interscapular brown fat with reduced BAT marker expression, accompanied by an impairment of insulin sensitivity. Although deletion of DGCR8 or Dicer results in different defects in WAT, both of them impair WAT browning upon exposure to cold [18,21]. These results demonstrate that miRNA plays a critical role in BAT development, WAT browning and brown/beige fat physiology.

It is interesting to introduce key miRNAs into DGCR8- or Dicer-knockout brown adipocytes and determine their sole contribution to brown adipocyte biology. Because global miRNA biogenesis has been blocked in DGCR8- or Dicer-knockout mice, these strains provide a clean background to identify the core set of miRNAs that are sufficient to support brown fat development. It has been reported that, in the Dicer-knockout brown adipocytes, reintroduction of *miR-346* and *miR-362* partially reverses the decrease in C/EBPβ (CCAAT/enhancer-binding protein β), whereas re-expression of *miR-365* increases *UCP1* mRNA expression 20-fold [21]. Another advantage of these platforms is to facilitate the identification of miRNA targets. Because mRNA targets are normally repressed by multiple miRNAs, the effect of a single miRNA is often masked by other miRNAs targeting the same mRNA. However, in the absence of other miRNAs, the effect of a given miRNA will be more evident when it is introduced back to DGCR8- or Dicer-knockout cells.

### Critical functional miRNAs in brown/beige adipocytes

miRNAs execute their regulatory function by participating in signalling networks and fine-tuning or switching signalling output levels [22]. Recent studies have identified several miRNAs functioning in different stages of brown/beige fat adipogenesis [23]; these regulatory networks are summarized as follows (Figure 2).

**Figure 2:**
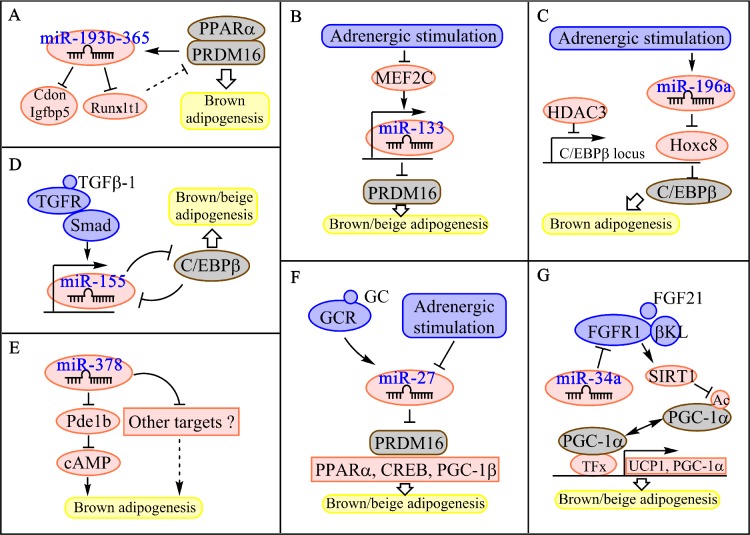
Key miRNA regulatory networks in brown/beige adipogenesis (**A**) Under the control of PRDM16, *miR-193b-365* promotes brown adipogenesis by targeting *Runx1t1*, *Cdon* and *Igfbp5*. (**B**) *miR-133*, transcriptionally controlled by MEF2, serves as a major negative regulator of brown/beige adipogenesis by directly targeting *PRDM16*. (**C**) *miR-196a* targets *Hoxc8* to up-regulate C/EBPβ for beige adipogenesis. HDAC3, histone deacetylase 3. (**D**) *miR-155* forms a bistable feedback loop with its target C/EBPβ and maintains the balance between proliferation and differentiation of brown pre-adipocytes. TGFR, TGF receptor; (**E**) *miR-378* promotes BAT differentiation and expansion partly through targeting *Pde1b* to elevate cAMP content. (**F**) *miR-27* targets several key brown transcription factors in response to both glucocorticoid (GC) signal and adrenergic-stimuli-induced brown/beige adipogenesis. CREB, cAMP-response-element-binding protein; GCR, glucocorticoid receptor. (**G**) *miR-34a* exerts roles in multiple tissues through targeting FGF signalling to up-regulate thermogenic genes such as those encoding PGC-1α and UCP1. Ac, acetyl; βKL, β-Klotho; SIRT1, sirtuin 1; TFx, thyroid receptor.

### miR-193b-365

Using microarray methods, researchers have identified a list of miRNAs enriched in BAT compared with WAT, among which *miR-193b* and *miR-365* are a bicistronic transcript [24]. The level of *miR-193b-365* is significantly up-regulated during brown adipogenesis. This miRNA cluster is needed for lineage-specific brown adipogenesis, as established by both overexpression and inhibition experiments. Blocking *miR-193b-365* causes a remarkable reduction in lipid accumulation during adipogenesis, whereas ectopic expression of *miR-193b* decreases mRNA levels of myogenic markers on C2C12 myoblasts and up-regulates mRNA levels of two master adipogenic factors, PPARγ and C/EBPα, thus inducing a transition of myoblasts to brown adipocytes under adipogenic differentiation conditions. *miR-193b* executes these function at least by directly repressing *Cdon* (cell adhesion-associated, oncogene-regulated), *Igfbp5* (insulin-like growth factor-binding protein 5) (two pro-myogenic factors) and *Runx1t1* (runt-related transcription factor 1 translocated to 1) (an inhibitor of brown adipogenesis) [25]. This regulatory network is initiated by the brown lineage determination factor PRDM16, as PRDM16 induces *miR-193b-365* expression at least partially by inducing expression of its transcription factor PPARα. Therefore *miR-193b-365* represents a new part of the PRDM16-induced programme. However, blocking *miR-193b-365* with miRNA inhibitors in mature brown adipocytes does not affect the expression of key brown adipocyte genes, indicating that this gene cluster carries out its primary function during development. Screening for miRNAs essential for maintenance of BAT features requires further investigation [24].

### miR-133

On the basis of profiling analysis of miRNAs in BAT before and after cold exposure, RNA sequencing for differentially expressed miRNAs between satellite cells and brown pre-adipocytes, and through bioinformatic analysis predicting PRDM16 targets, several groups have successively revealed *miR-133* as a critical regulator of adipocyte browning [26–28].

*miR-133* belongs to a group of myomiRs (muscle-specific miRNAs) and is a potent repressor of non-muscle genes, thereby playing a role in embryonic stem cell differentiation and muscle specification [29,30]. *miR-133* serves as a major negative regulator of brown/beige adipogenesis. It has a distinct expression pattern among myogenic and brown adipogenic lineages, and their progenitors. The level of *miR-133* is down-regulated during the induction of brown adipocyte differentiation and in BAT after cold exposure [26,27]. The down-regulation of *miR-133* is inversely correlated with PRDM16 and UCP1 expression levels. Functional assays for miRNA target identification reveal that both *miR-133a* and *miR-133b* directly target the conserved 8-nt seed sequences in the 3′UTR of *PRMD16* mRNA [26–28]. Moreover, overexpression of *miR-133* in brown pre-adipocytes dramatically decreases the mRNA level of PRDM16, as well as other brown adipogenic markers and general adipogenic markers [27], but increases myogenic markers [26]. By contrast, inhibition of *miR-133* causes an increase in BAT transcription factors PPARγ and PPARα, and a decrease in myogenic markers [26]. Therefore the brown lineage commitment is influenced directly by *miR-133* through targeting PRDM16. *miR-133* is regulated directly by MEF2 (myocyte enhancer factor 2) during adrenergic-stimuli-induced BAT development and differentiation. Adrenergic stimulation upon cold exposure leads to a decrease in MEF2, resulting in a reduced expression of *miR-133* and an increased expression of PRDM16, which facilitates brown lineage commitment [26].

Inhibition of *miR-133* during BAT differentiation causes enhanced mitochondrial activity and BAT programme, leading to an increase in the basal oxygen consumption rate [26]. Similar modulations are observed in the progression of frozen injury-induced muscle regeneration in which *miR-133* antagonism induces an increase in uncoupled respiration, glucose uptake and thermogenesis in intramuscular brown adipocytes [27]. The bioenergetic effects of *miR-133* antagonism on muscle cells make it a critical modulator of whole-body energy expenditure: antagonism of *miR-133* in mice fed with either a normal diet or an HFD (high-fat diet) display a significant increase in total energy expenditure, without much change in physical activity and food intake. Moreover, antagonism of *miR-133* leads to improved glucose tolerance and decreased infiltration of inflammatory cells in eWAT (epididymal WAT) [27]. Consistent with this, knockout of *miR-133* in mice leads to SAT (subcutaneous adipose tissue) browning and increased insulin sensitivity [28]. All of these studies indicate that *miR-133* can serve as a therapeutic target to defend against diet-induced obesity.

### miR-196a

*miR-196a* expression is induced in SVF (stromal-vascular fraction) cells during brown adipogenesis by an adenylate cyclase activator, forskolin, or cold exposure [31]. The *miR-196a* gene is located within the gene clusters encoding the Hox (homeobox) family, which plays a role in system differentiation including adipogenesis [31,32]. One of the Hox genes, *Hoxc8*, is categorized as a white-fat gene and a repressor of brown adipogenesis [33]. Hoxc8 targets the C/EBPβ regulatory sequence and represses C/EBPβ, a positive regulator of the BAT programme. During the brown adipogenesis of white fat progenitors, the expression of *Hoxc8* is suppressed directly by *miR-196a*, which facilitates the browning progression. In iBAT (interscapular BAT)-SVF cells, *miR-196a* is barely expressed, and its target *Hoxc8* is even absent, which implies that *miR-196a* does not play a role in conventional brown adipogenesis [31]. Fat-specific *miR-196a* transgenic mice generate metabolically functional beige cells in WAT, and exhibit enhanced energy expenditure, improved glucose metabolism and resistance to obesity. Therefore manipulation of *miR-196a*–Hoxc8–C/EBPβ signalling in WAT progenitors to initiate browning can be a therapeutic strategy for obesity-associated metabolic consequences.

### miR-155

TGFβ_1_ (transforming growth factor β1) potently inhibits adipogenesis, and knockout of Smad3 induces the WAT to BAT phenotypic transition [34,35]. A recent study demonstrated that the inhibitory effect of TGFβ_1_/Smad signalling on brown fat adiposeness is partly exerted through induction of *miR-155* [36]. *miR-155* is highly expressed in pre-adipocytes, but decreases dramatically after induction of differentiation. It forms a bistable feedback loop with its target C/EBPβ to maintain the balance between proliferation and differentiation of brown pre-adipocytes. *miR-155* transgenic mice display reduced BAT mass, whereas *miR-155*-knockout mice show a better adaption to cold exposure by recruiting brite fat cells (inducible beige cells) and enhancing differentiation of BAT [36]. These data demonstrate that *miR-155* serves as a negative regulator for brown fat differentiation.

### miR-27

A single miRNA family can regulate a large number of targets, some of which may be involved in the same biological process or signalling pathway [16,23]. *miR-27a*/*miR-27b* is such a miRNA family, which serves as a upstream regulator of brown/beige adipogenesis by directly targeting several key transcription factors such as PRDM16, PPARα, CREB (cAMP-response-element-binding protein) and PGC-1β [37]. Moreover, antagonizing *miR-27b* function prevents dexamethasone (one type of glucocorticoid) induced inhibition of BAT differentiation in primary adipocytes, and improves glucocorticoid treatment-induced central fat accumulation and metabolic dysfunction in mice. Glucocorticoid induces the expression of *miR-27b* through glucocorticoid receptor-mediated transcriptional regulation [38]. Therefore *miR-27* is a responder of glucocorticoid signal, as well as a central regulator of brown/beige adipogenesis.

### miR-378

*miR-378* resides in the gene locus of PGC-1β and is induced during brown adipogenesis. *miR-378* promotes BAT differentiation and expansion, but suppresses the formation of beige adipocytes in subcutaneous WAT. *miR-378* directly targets *Pde1b* (phosphodiesterase 1b) that catalyses the turnover of cAMP [39]. This may be one of the regulators in BAT activity and expansion, as single nucleotide polymorphisms in the *Pde1b* gene are associated with cattle back-fat thickness, and cAMP can potentiate adipocyte differentiation [40,41]. Indeed, *miR-378* transgenic mice display elevated cAMP levels which leads to increased lipolysis and activity in BAT, accompanied with enlarged organ mass and improved metabolic profile.

### miR-34a

Upon cold exposure, 3T3-L1 fat cells display a marked decrease in *miR-34a* and an increase in browning-related genes, such as those encoding UCP1, PGC-1α and PRDM16. This inverse correlation is also observed in adipose tissue, which suggests that *miR-34a* inhibits fat browning [42]. Mechanistically, *miR-34a* directly targets FGFR1 (fibroblast growth factor receptor 1) to attenuate FGF21 (fibroblast growth factor 21)/SIRT1 (sirtuin 1)-dependent deacetylation of PGC-1α, which controls the browning transcriptional programme [42,43]. Circulating FGF21, an endocrine mitokine, enhances fatty acid β-oxidation in liver and adipose tissue, induces WAT browning, and promotes protection from diet-induced obesity and insulin resistance [44]. The integrated actions from multiple tissues induced by FGF21 signalling makes its upstream *miR-34a* a potent modulator in metabolic regulatory networks. Importantly, beneficial effects mediated by inhibition of *miR-34a* in HFD-fed mice include several aspects, including reduced adiposity and improved serum profiles [42]; *miR-34a* inhibition induces browning in all types of WAT, and promotes a more robust BAT programme [42]. These distinctive effects imply that *miR-34a* exerts roles in multiple tissues through targeting FGF signalling.

## FUNCTION OF lncRNAs IN BROWN/BEIGE FAT

The function of lncRNAs in BAT is poorly understood despite existing reports on their expression pattern in BAT [45,46]. Using massively parallel sequencing of polyadenylation-selected RNAs, researchers have identified 175 lncRNAs which are regulated during adipogenesis. Further RNAi-mediated loss-of-function experiments have characterized ten lncRNAs that are required for adipogenesis and are referred to as lnc-RAP-*n* (lncRNA Regulated in AdiPogenesis) [47]. One of them, lnc-RAP-1 (or Firre, functional intergenic repeating RNA element) interacts with the nuclear matrix factor hnRNPU (heterogeneous nuclear ribonucleoprotein U) to mediate *trans*-chromosomal interactions, and brings loci encoding known regulators of adipogenesis into close proximity. This hnRNPU–Firre-mediated interaction may be required for proper function of multiple biological processes including adipogenesis [48].

This study is mainly based on the WAT system, but the role of lncRNA in brown adipocyte differentiation is not well understood. A recent research has identified Blnc1 (brown fat lncRNA 1) as a BAT-enriched lncRNA, which promotes brown and beige adipocyte differentiation through a ribonucleoprotein complex formed with EBF2 (early B-cell factor 2) to enhance expression of thermogenic genes such as UCP1. This regulation pattern is feedforward because Blnc1 itself is positively regulated by EBF2 [49].

These previous studies suffer from an incomplete annotation of lncRNA in adipose tissue. To address this obstacle, our group performed deep RNA-seq from BAT, inguinal WAT and eWAT, which provides us with approximately half a billion of reads for transcriptome reconstruction. We developed a computational pipeline and identified more than 1500 lncRNAs expressed in adipose tissue. Of these, 127 are BAT-restricted, and many of them are targeted by key adipogenic regulators such as PPARγ and C/EBPα [50]. Since they are BAT-enriched, we refer to them as lnc-BATEs. lnc-BATE1 is up-regulated 30-fold during adipogenesis and is required for brown adipogenesis and the maintenance of brown adipocyte features. lnc-BATE1 specifically binds the nuclear matrix factor hnRNPU; both of them are required for proper brown adipogenesis [48,50], but how this ribonucleotide complex exerts its functions is not well understood and requires further investigation.

Manipulation of BAT and WAT browning has been linked to a healthy metabolic phenotype and a defence against obesity and the metabolic syndrome. Several recent studies have identified new BAT-specific lncRNAs and revealed that some of them, such as lnc-BATE1 and Blnc1, promote brown and/or beige adipocyte differentiation and physiological function (Figure 3). Although the detailed regulatory networks and mechanisms are still not fully understood, lncRNAs are promising therapeutic targets for obesity and associated metabolic diseases.

**Figure 3:**
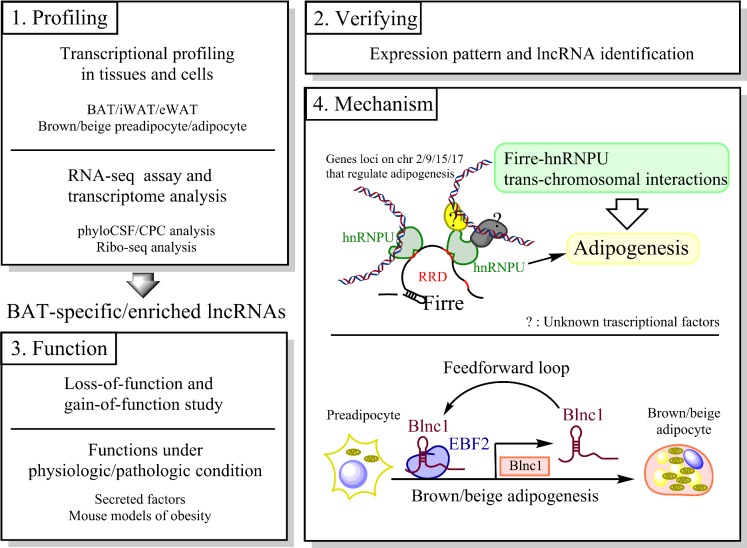
lncRNAs in BAT adipogenesis The research route for exploiting lncRNAs in BAT adipogenesis contains four steps: 1, transcriptional profiling; 2, verifying lncRNAs; 3, functional study; and 4, mechanism exploring. Two examples for regulatory mechanisms of lncRNAs in BAT are presented (upper panel: Firre–hnRNPU interplay-mediated *trans*-chromosomal interactions promotes adipogenesis; lower panel: Blnc1 *cis*-regulates EBF2, and Blnc1–EBF2 forms a feedforward loop to promote brown/beige adipogenesis). iWAT, inguinal WAT.

## CONCLUSIONS AND PERSPECTIVES

BAT protects against obesity by promoting energy expenditure via uncoupled respiration, and is regarded as an attractive therapeutic target organ for obesity and its pathological consequences [1]. In the present review, we have summarized the functional role of two types of intrinsic factor, miRNA and lncRNA, in brown/beige adipogenesis and physiological function. Increased expression of *miR-193b-365*, *miR-196a* and *miR-378* or inhibition of *miR-133*, *miR-155*, *miR-27* and *miR-34a* can promote brown fat adipogenesis. Although it is still insufficient to construct a whole miRNA regulatory network in BAT adipogenesis, these studies display explicit correlations among miRNAs themselves and their targeted signalling pathways. On this basis, further investigations will probably reveal the core set of miRNAs responsible for reconstructing BAT with higher activities.

Comparing with the relatively unifying molecular mechanism of miRNAs, the mechanisms used by lncRNAs are more diverse. Firre and lnc-BATE1 interact with nuclear matrix factor hnRNPU to make a spatial reconstruction of chromosome to bring a few metabolic genes into closer proximity which may lead to co-regulation [48]. EBF2 and its *cis*-regulator Blnc1 form a feedforward loop to promote brown/beige adipogenesis [49]. More functional lncRNAs with different mechanisms are expected to emerge in the near future. These lncRNA studies, together with the miRNA study, will depict a new regulatory layer governing brown/beige fat plasticity under physiological and pathological conditions.

## Abbreviations

BAT: brown adipose tissue
Blnc1: brown fat lncRNA 1
C/EBP: CCAAT/enhancer-binding protein
CtBP: C-terminal binding protein
DGCR8: DiGeorge syndrome critical region 8
EBF2: early B-cell factor 2
eWAT: epididymal WAT
FGF: fibroblast growth factor
Firre: functional intergenic repeating RNA element
HFD: high-fat diet
hnRNPU: heterogeneous nuclear ribonucleoprotein U
Hox: homeobox
lnc-BATE: lncRNA BAT-enriched
lnc-RAP-*n*: lncRNA Regulated in AdiPogenesis
lncRNA: long non-coding RNA
MEF2: myocyte enhancer factor 2
ncRNA: non-coding RNA
PGC: PPARγ co-activator
PPAR: peroxisome-proliferator-activated receptor
PRDM16: PR domain-containing 16
SAT: subcutaneous adipose tissue
SVF: stromal-vascular fraction
TGFβ_1_: transforming growth factor β1
UCP1: uncoupling protein 1
WAT: white adipose tissue
